# Supplementary material for: The neocortical infrastructure for language involves region-specific patterns of laminar gene expression
Source: Proc Natl Acad Sci U S A. 2024 Aug 12;121(34):e2401687121. doi: 10.1073/pnas.2401687121 (PMC11348331; doi:10.1073/pnas.2401687121)
Supplement: Supplementary file 1 — Appendix 01 (PDF) [file pnas.2401687121.sapp.pdf]

Supplementary Information

Wong & Sha et al., The neocortical infrastructure for language involves region-specific patterns of laminar gene expression

Contents:

- Supplementary Figures 1-8 on pages 2-9.
- Supplementary Tables 1-6 on pages 10-15.

(Datasets S1-S5 are within separate files.)

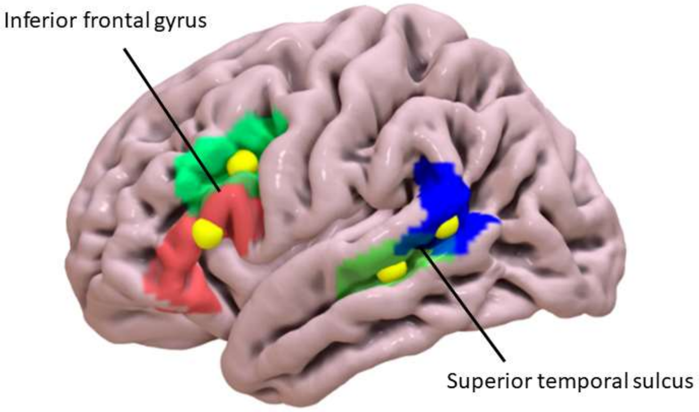

Supplementary Figure 1. Schematic of the left-hemisphere regions sampled in this study. Two tissue blocks were taken from the inferior frontal gyrus. Blocks were approximately centred on the yellow spots indicated (block 'gfi1' more anterior/inferior, block 'gfi3' more posterior/superior). Two tissue blocks were also taken from the superior temporal sulcus. Again, blocks were approximately centred on the yellow spots indicated (block 'gts4' more anterior/inferior, block 'gts5' more posterior/superior). The broader coloured regions around the yellow spots represent four areas defined in the SENtence Supramodal Areas AtlaS (SENSAAS) (Labache et al. (2019) – see reference in the main manuscript).

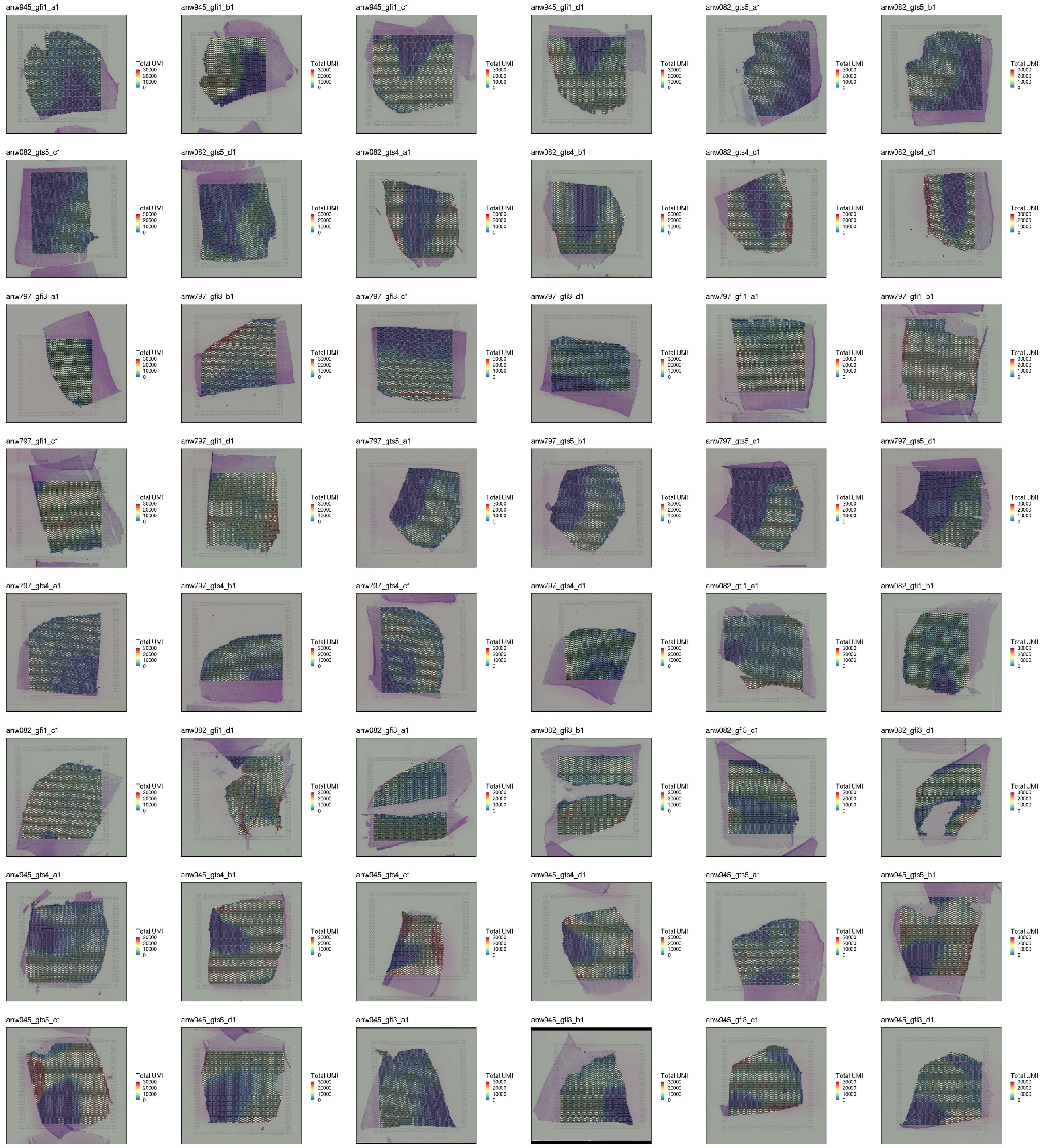

Supplementary Figure 2. Unique molecular identifier counts across all spots for 48 cortical tissue sections. The pink/purple areas were outside of the capture area for spatial transcriptomics. The labels of the 48 cortical tissue sections are given as Donor_Block_Section, where the three donors were anw082, anw797 and anw945, the blocks were gfi1 & gf13 from the inferior frontal gyrus and gts4 & gts5 from the superior temporal sulcus, and the tissue sections from each block are called a1 & a2 (the first adjacent pair of sections) and a3 & a4 (second pair of adjacent sections).

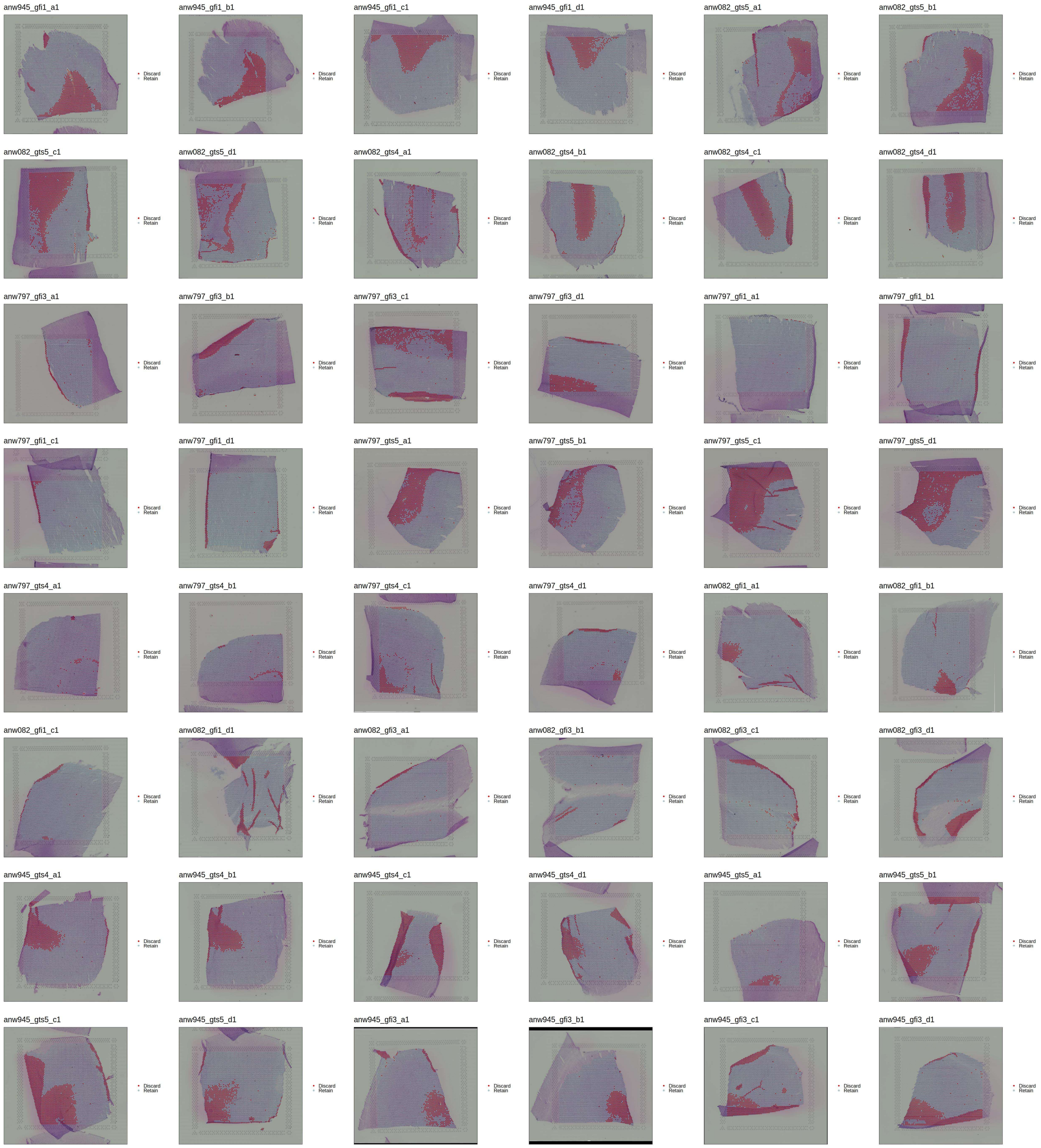

Supplementary Figure 3. Spots excluded during spot-level quality control. Spots coloured red were excluded according to the process described in the main text (Methods). The labels of the 48 cortical tissue sections are given as Donor_Block_Section, where the three donors were anw082, anw797 and anw945, the blocks were gfi1 & gf13 from the inferior frontal gyrus and gts4 & gts5 from the superior temporal sulcus, and the tissue sections from each block are called a1 & a2 (the first adjacent pair of sections) and a3 & a4 (second pair of adjacent sections).

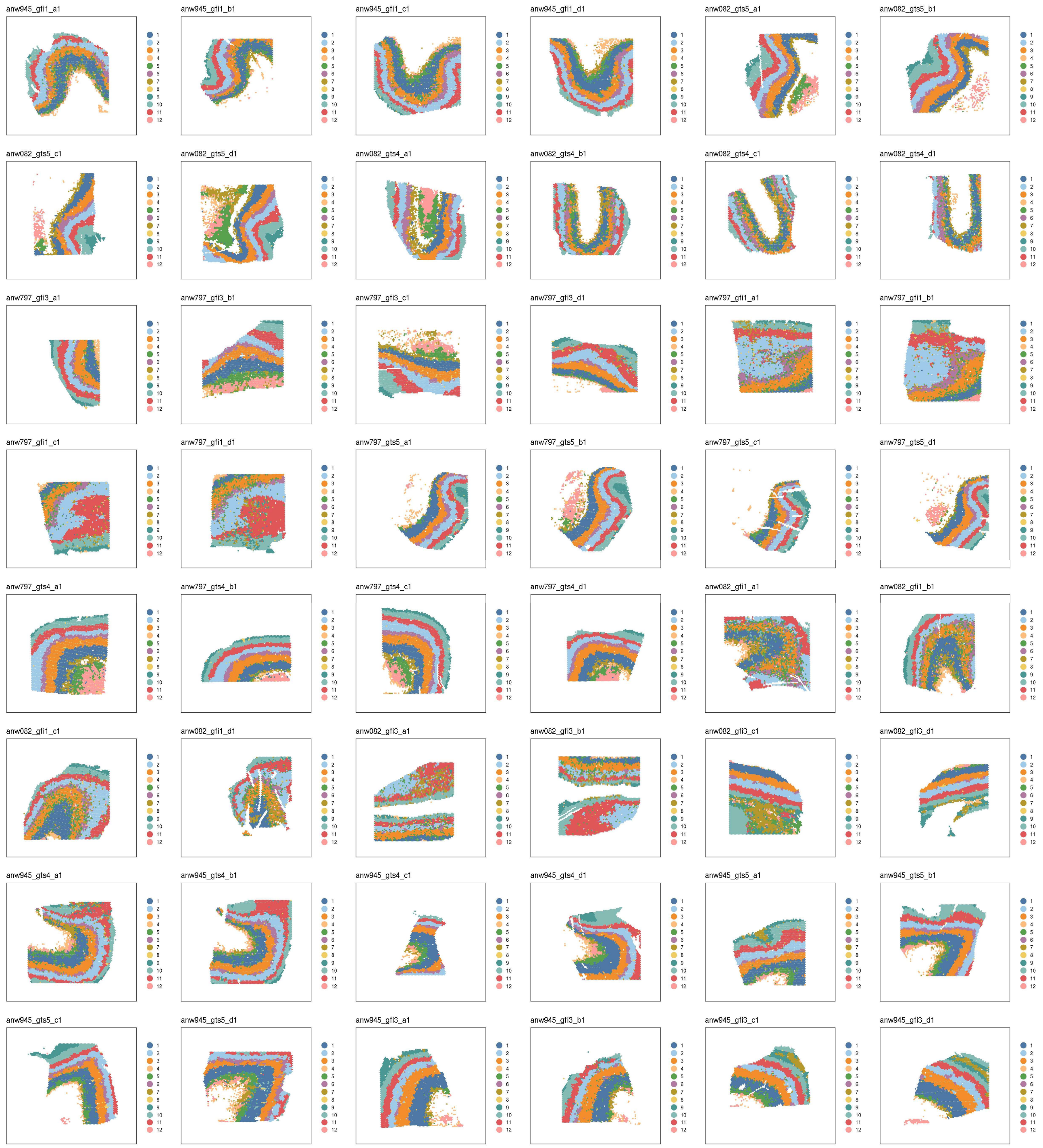

Supplementary Figure 4. Spatial distributions of gene expression data-driven clusters of spots across 48 tissue sections. The labels of the 48 cortical tissue sections are given as Donor_Block_Section, where the three donors were anw082, anw797 and anw945, the blocks were gfi1 & gf13 from the inferior frontal gyrus and gts4 & gts5 from the superior temporal sulcus, and the tissue sections from each block are called a1 & a2 (the first adjacent pair of sections) and a3 & a4 (second pair of adjacent sections).

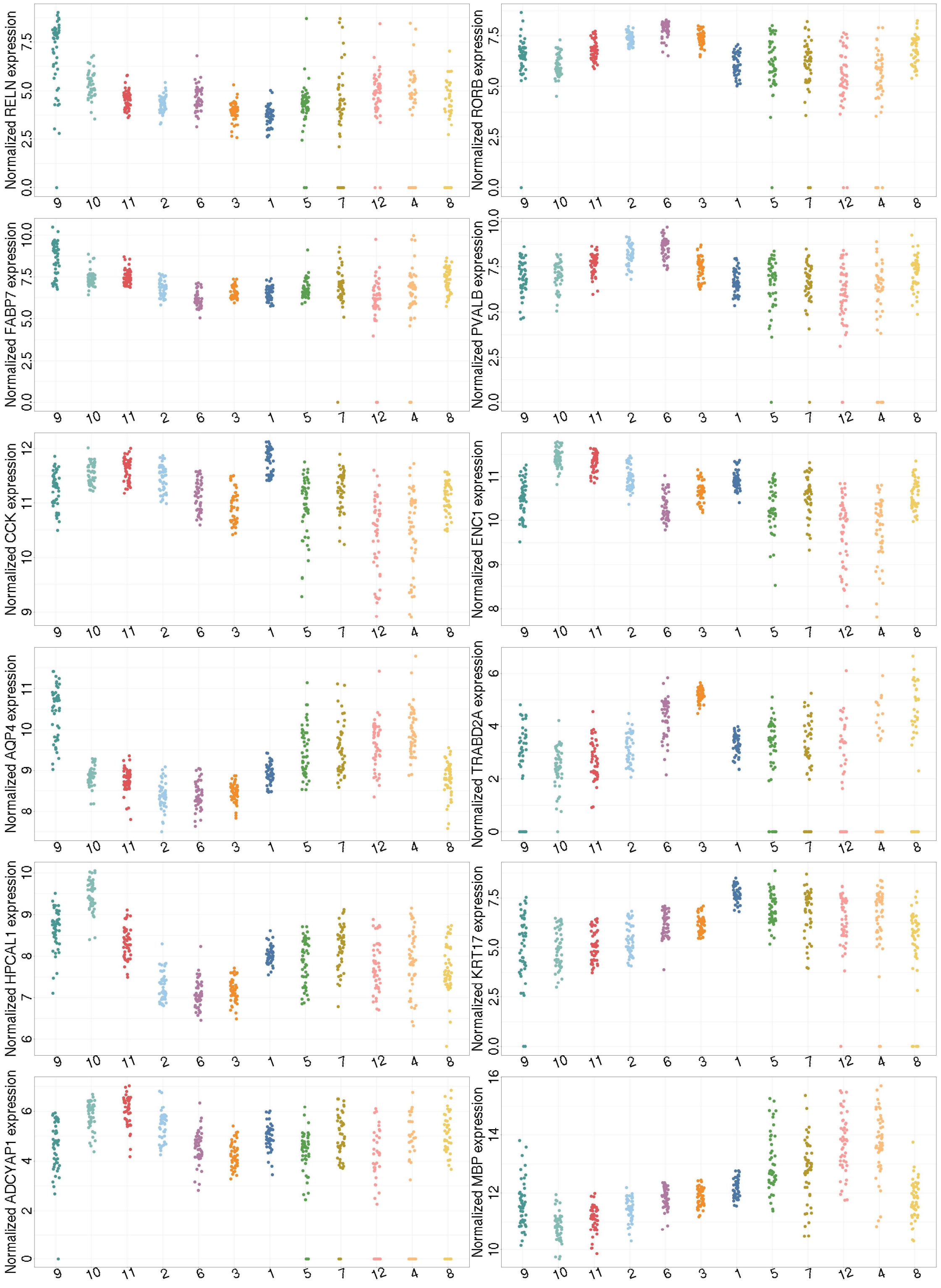

Supplementary Figure 5. The expression levels of layer marker genes in data-driven clusters. Each panel shows the expression of a single marker gene, with the x-axis showing the data-driven clusters and the y-axis showing the normalized gene expression across 48 tissue sections. Clusters are ordered from left-to-right according to their laminar spatial locations from upper to lower. These data support the following correspondence: layerI=cluster9, layerII=cluster10, layerIII=clusters11&2, layerIV=cluster 6, layerV=cluster 3, layerVI=cluster1, with other clusters corresponding to white matter or with sporadic spatial distributions.

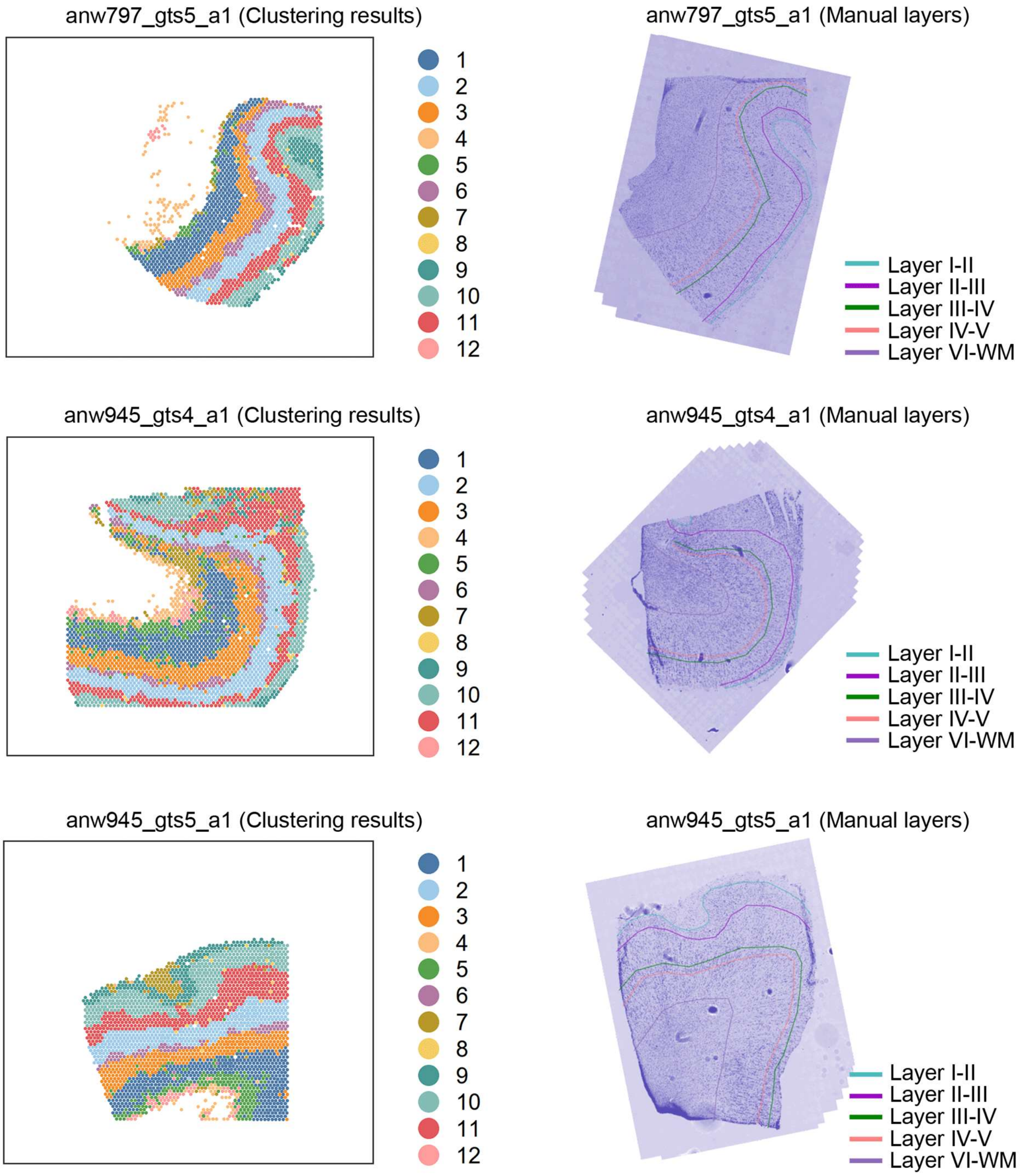

Supplementary Figure 6. Cluster-layer correspondence assessed through cytoarchitecture. Comparison between data-driven gene expression clusters from the spatial transcriptomic data, and manually-defined cortical layers based on cytoarchitecture, across three pairs of tissue sections.

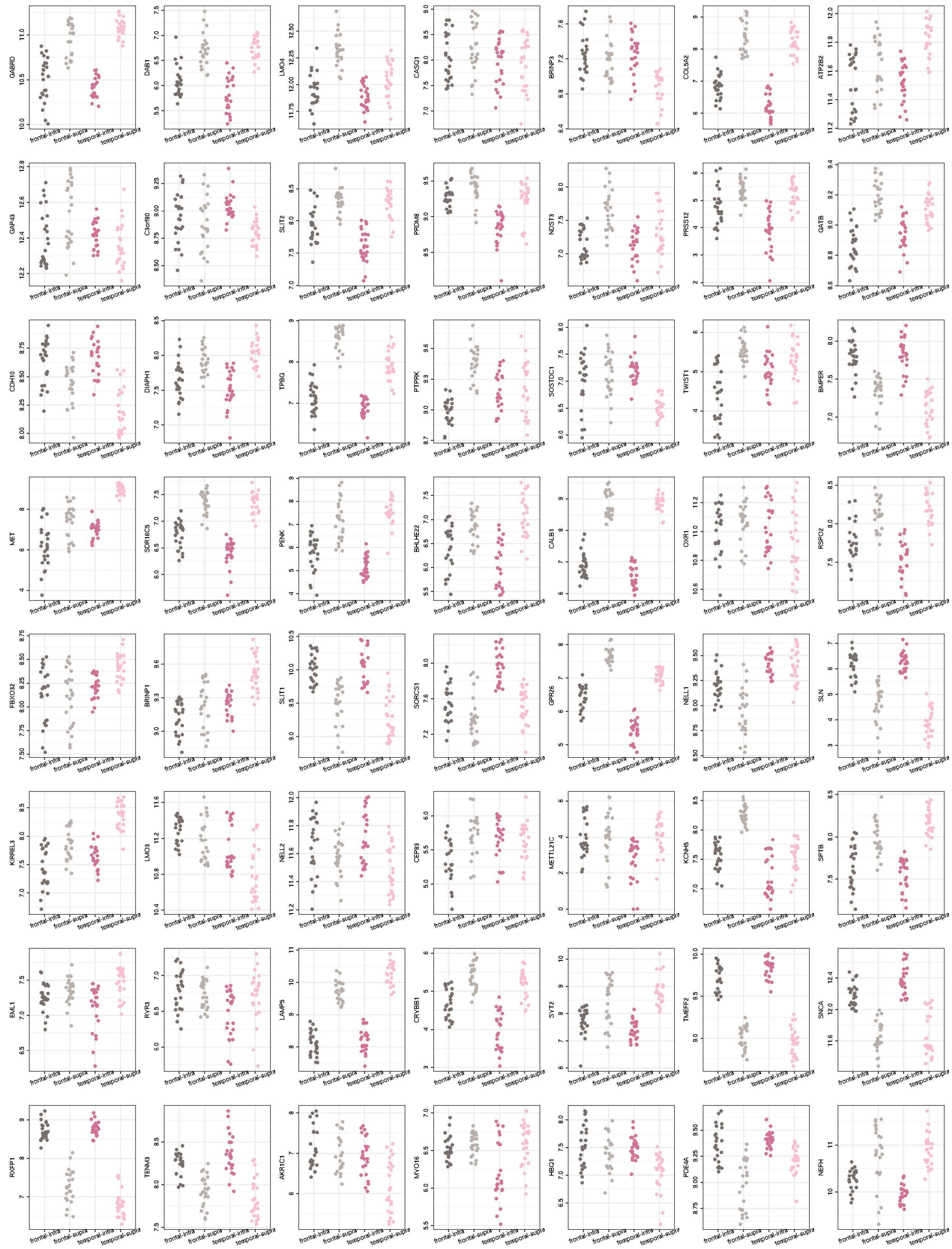

Supplementary Figure 7. Significant layer*lobe interaction effects for 56 genes that also showed upregulation in Layer II/III excitatory neurons and/or Layer V/VI cortico-cortical projection neurons. Each panel shows the expression of a single gene, with the x-axis showing four pseudo-bulked clusters (see main text) and the y-axis showing the normalized gene expression across 48 tissue sections.

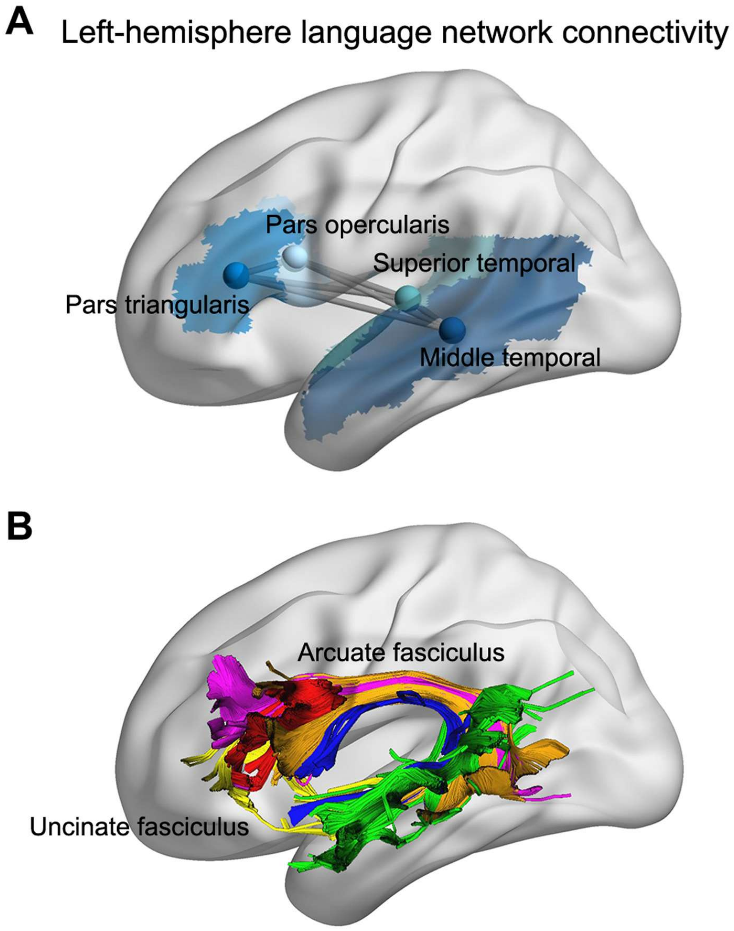

Supplementary Figure 8. White matter connectivity between four regions of the core left-hemisphere language network. (A) The four cortical regions are shown in different shades of blue, as defined in the Automated Anatomical Labelling brain atlas (Tzourio-Mazoyer et al. 2002), and applied in the genome-wide association study of Sha et al. (2023) in 30,810 adults (see reference list in the main manuscript). Also shown is an abstract network representation where the four cortical regions are nodes and the structural connections between them are edges. In the present study we were interested in the four frontal-temporal network edges, i.e. pars opercularis - superior temporal cortex; pars triangularis - superior temporal cortex; pars opercularis - middle temporal cortex; and pars triangularis - middle temporal cortex. (B) Visualization of the white matter connections between the four cortical regions in an example individual, with gold representing connections between the pars opercularis and middle temporal cortex, blue representing connections between the pars opercularis and superior temporal cortex, purple representing connections between the pars triangularis and middle temporal cortex, and yellow representing connections between the pars triangularis and superior temporal cortex. Also shown are connections between the pars opercularis and pars triangularis (red), and connections between the middle temporal cortex and superior temporal cortex (green), but these within-lobe connections were not considered in the present study. Figure reproduced from Sha et al, (2023) under an open access Creative Commons Attribution License 4.0 (CC BY) (see reference list in the main manuscript).

**Supplementary Table 1. Information for three neurotypical donors**					
Donor ID	Age (years)	Sex	RNA integrity number	Post mortem delay to autopsy (hours:minutes)	ABC scores*
ANW082	63	Female	7	08:10	A0 B0 C0
ANW797	59	Male	8.8	08:00	A1 B1 C0
ANW945	59	Female	8.5	08:10	A0 B0 C0
*According to Hyman et al. (2012), National Institute on Aging–Alzheimer's Association guidelines for the neuropathologic assessment of Alzheimer's disease. Alzheimer's & Dementia, 8: 1-13 (see reference list in the main manuscript).					

Supplementary Table 2: Gene Ontology enrichment analysis of 72 genes that showed significant layer-by-lobe interaction effects. Sets with adjusted enrichment p values <0.01 are shown.					
Gene set	P-value	FDR=adjusted p-value	Odds Ratio	Combined score	Driver genes
Retinal Ganglion Cell Axon Guidance (GO:0031290)	4.45E-07	0.0002166	433.17	6335.24	NELL1, PTPRM, SLIT2
Neuron Projection Extension Involved In Neuron Projection Guidance (GO:1902284)	8.88E-07	0.0002166	288.77	4023.87	NELL1, SLIT1, SLIT2
Axon Extension Involved In Axon Guidance (GO:0048846)	8.88E-07	0.0002166	288.77	4023.87	NELL1, SLIT1, SLIT2
Regulation Of Endothelial Cell Migration (GO:0010594)	1.631E-05	0.002985	17.84	196.7	GLUL, PTPRM, BMPER,NELL1, SLIT2
Negative Regulation Of Cellular Process (GO:0048523)	2.155E-05	0.003155	5.94	63.8	SLIT2, SLIT1, PTPRK, WFS1, NACC2, GNG4
Metal Ion Transport (GO:0030001)	3.505E-05	0.004276	10.89	111.71	CALCRL, RAMP1, RYR3, ATP2B2, SLN, SCN7A
Calcium Ion Transport (GO:0006816)	5.224E-05	0.005463	13.82	136.3	CALCRL, RAMP1, RYR3, ATP2B2, SLN
Axon Extension (GO:0048675)	7.523E-05	0.006883	43.28	410.93	NELL1, SLIT1, SLIT2
Regulation Of Catecholamine Secretion (GO:0050433)	0.000123	0.007741	36.06	324.65	SYT2, SNCA, HRH3
Negative Regulation Of Cellular Response To Growth Factor Stimulus (GO:0090288)	0.000125	0.007741	17.44	156.71	SOSTDC1, BMPER, SLIT2
Regulation Of Retinal Ganglion Cell Axon Guidance (GO:0090259)	0.0001269	0.007741	189.76	1702.55	SLIT1, SLIT2
Negative Regulation Of Chemokine-Mediated Signaling Pathway (GO:0070100)	0.0001269	0.007741	189.76	1702.55	SLIT1, SLIT2
Regulation Of Catecholamine Secretion (GO:0050433)	0.000123	0.007741	36.06	324.65	CALCR1, RAMP1

**Supplementary Table 3: Gene Ontology enrichment analysis of 56 genes upregulated in LII/III and/or LV/VI excitatory neurons that showed significant layer-by-lobe interaction effects. Gene ontology sets with FDR-adjusted enrichment p values <0.01 are shown.**					
Gene set	P-value	FDR-adjusted p-value	Odds Ratio	Combined score	Driver genes
Neuron Projection Extension Involved In Neuron Projection Guidance (GO:1902284)	8.23E-10	2.76E-07	767	16044.12	NELL1, NELL2, SLIT1, SLIT2
Axon Extension Involved In Axon Guidance (GO:0048846)	8.23E-10	2.76E-07	767	16044.12	NELL1, NELL2, SLIT1, SLIT2
Axon Extension (GO:0048675)	4.69E-07	0.0001047	80.67	1175.54	NELL1, NELL2, SLIT1, SLIT2
Regulation Of Retinal Ganglion Cell Axon Guidance (GO:0090259)	0.00007659	0.00733	246.19	2333.12	NELL1, SLIT2
Retinal Ganglion Cell Axon Guidance (GO:0031290)	0.00007659	0.00733	246.19	2333.12	SLIT1, SLIT2
Negative Regulation Of Chemokine-Mediated Signaling Pathway (GO:0070100)	0.00007659	0.00733	246.19	2333.12	SLIT1, SLIT2
Axon Guidance (GO:0007411)	0.00006035	0.00733	13.48	130.97	GAP43, NELL1, NELL2, SLIT1, SLIT2
Semaphorin-Plexin Signaling Pathway (GO:0071526)	0.000117	0.008186	36.36	329.18	NELL1, NELL2, MET
Regulation Of Endothelial Cell Migration (GO:0010594)	0.0001079	0.008186	18.19	166.12	BMPER, NELL1, NELL2, SLIT2
Neuron Differentiation (GO:0030182)	0.0001222	0.008186	11.54	103.98	ATP2B2, BHLHE22, BRINP3, MET, TENM3
Regulation Of Chemokine-Mediated Signaling Pathway (GO:0070099)	0.0001603	0.008223	147.7	1290.68	SLIT1, SLIT2
Sympathetic Ganglion Development (GO:0061549)	0.0002133	0.008223	123.07	1040.33	NELL1, NELL2
Negative Regulation Of Transporter Activity (GO:0032410)	0.0002133	0.008223	123.07	1040.33	TWIST1, SNCA
Response To Magnesium Ion (GO:0032026)	0.0002133	0.008223	123.07	1040.33	RYR3, SNCA
Aortic Valve Morphogenesis (GO:0003180)	0.000151	0.008223	33.15	291.63	SLIT1, SLIT2, TWIST1
Aortic Valve Development (GO:0003176)	0.0001909	0.008223	30.45	260.81	SLIT1, SLIT2, TWIST1
Branching Morphogenesis Of An Epithelial Tube (GO:0048754)	0.0002209	0.008223	28.89	243.19	MET, NELL1, SLIT2
Axonogenesis (GO:0007409)	0.0001802	0.008223	10.59	91.27	NEFH, NELL1, NELL2, SLIT1, SLIT2
Motor Neuron Axon Guidance (GO:0008045)	0.0002737	0.009653	105.49	865.35	SLIT1, SLIT2

Supplementary Table 4. Association results for 56 genes in relation to white matter connectivity in 30,810 adults

Structural connectivity	56-gene set association P value (GAUSS)
Pars opercularis - superior temporal	0.21
Pars opercularis - middle temporal	0.14
Pars triangularis - superior temporal	0.02
Pars triangularis - middle temporal	0.004

		Pars opercularis - superior temporal		Pars opercularis - middle temporal		Pars triangularis - superior temporal		Pars triangularis - middle temporal	
GENE	Chromosome	Z	P	Z	P	Z	P	Z	P
AKR1C1	10	1.6738	0.047089	0.29633	0.38349	0.87686	0.19028	0.083066	0.4669
ATP2B2	3	-0.11215	0.54465	0.77731	0.21849	1.0032	0.15789	1.1063	0.1343
BHLHE22	8	-0.030563	0.51219	-1.0539	0.85404	1.7546	0.039661	3.0301	0.0012223
BMPER	7	1.3204	0.093351	1.2053	0.11404	-0.0060335	0.50241	-1.3281	0.90793
BRINP1	9	-1.1742	0.87983	1.2547	0.10479	-2.7303	0.99684	-1.2872	0.901
BRINP3	1	-0.7042	0.75935	0.98202	0.16305	-0.27759	0.60934	2.8849	0.0019579
C3orf80	3	-0.0507	0.52022	-0.31364	0.6231	-0.71773	0.76354	1.0433	0.1484
CALB1	8	1.8579	0.031595	0.46313	0.32163	-0.32167	0.62615	0.014893	0.49406
CASQ1	1	-0.68139	0.75219	-0.37662	0.64677	0.41362	0.33958	0.76813	0.2212
CDH10	5	0.093885	0.4626	-1.3261	0.9076	-1.1016	0.86469	0.16563	0.43422
CEP83	12	0.19346	0.4233	0.54956	0.29131	1.1444	0.12623	-0.11277	0.5449
COL5A2	2	0.48276	0.31463	1.5187	0.064422	0.37082	0.35539	1.8618	0.031318
CRYBB1	22	0.37343	0.35441	-0.71126	0.76154	-0.079692	0.53176	-0.011568	0.50461
DAB1	1	1.3682	0.085618	0.56457	0.28618	0.036446	0.48546	1.0901	0.13784
DIAPH1	5	1.0452	0.14796	0.52807	0.29872	1.5536	0.060145	-0.70744	0.76035
EML1	14	0.67017	0.25138	0.34053	0.36673	1.3241	0.092727	0.80545	0.21028
FBXO32	8	-0.87752	0.8099	0.58595	0.27895	0.53145	0.29755	0.50818	0.30566
GABRD	1	2.5581	0.0052623	-1.0544	0.85414	0.18203	0.42778	1.1325	0.12871
GAP43	3	-1.9555	0.97474	2.647	0.0040603	1.5766	0.057438	1.1021	0.13521
GATB	4	-0.56554	0.71415	1.0883	0.13823	0.66545	0.25288	-0.57994	0.71902
GPR26	10	0.11783	0.4531	-0.73998	0.77034	-0.48391	0.68578	1.267	0.10258
HBQ1	16	0.23013	0.409	-1.5167	0.93533	1.512	0.065272	-1.67	0.95254
KCNH5	14	0.022647	0.49097	1.1599	0.12303	0.18229	0.42768	0.19096	0.42428
KIRREL3	11	-0.022237	0.50887	-1.7295	0.95814	-0.7797	0.78222	-1.5613	0.94077
LAMP5	20	-0.93097	0.82407	0.55291	0.29016	1.5013	0.066633	0.055918	0.4777
LMO3	12	0.025129	0.48998	2.2373	0.012632	0.52843	0.2986	0.88881	0.18705
LMO4	1	0.36786	0.35649	2.0103	0.022199	0.28124	0.38926	1.1906	0.1169
MET	7	-0.61258	0.72992	-0.062044	0.52474	1.2827	0.099801	1.4032	0.080282
METTL21C	13	-0.15551	0.56179	1.6823	0.046255	-1.1531	0.87557	0.90891	0.1817
MYO16	13	-0.67618	0.75054	-0.16699	0.56631	-0.13547	0.55388	-0.15204	0.56042
NDST3	4	1.2947	0.09772	0.78678	0.2157	-0.45134	0.67413	-0.25512	0.60069
NEFH	22	-0.46866	0.68034	0.82876	0.20362	1.1766	0.11968	0.012983	0.49482
NELL1	11	1.1088	0.13375	-1.6986	0.9553	0.39156	0.34769	0.53762	0.29542
NELL2	12	-0.014197	0.50566	-1.1881	0.88259	-0.27551	0.60854	2.2263	0.012997
OXR1	8	1.4079	0.079573	-1.0342	0.84949	0.4279	0.33436	0.72871	0.23309
PDE4A	19	1.585	0.056483	-1.8423	0.96728	0.4756	0.31718	0.23352	0.40768
PENK	8	-0.84202	0.80011	-1.4063	0.92018	1.7719	0.038208	0.28896	0.38631
PRDM8	4	-0.33508	0.63122	1.6771	0.046759	1.0544	0.14585	1.2074	0.11364
PRSS12	4	1.5441	0.061286	1.5554	0.059924	-0.19319	0.5766	1.4794	0.069517
PTPRK	6	0.59829	0.27482	0.2748	0.39173	0.81865	0.20649	0.46324	0.3216
RSPO2	8	-0.7712	0.77971	-0.011299	0.50451	1.8468	0.032385	1.0518	0.14645
RXFP1	4	0.72571	0.23401	0.72732	0.23351	1.0452	0.14796	0.41792	0.338
RYR3	15	-0.049548	0.51976	0.33078	0.37041	0.79057	0.2146	1.8003	0.035908
SDR16C5	8	-1.3018	0.90351	-0.036295	0.51448	1.5758	0.057541	-0.99206	0.83942
SLIT1	10	-1.1152	0.86762	1.5275	0.063312	2.1826	0.014534	2.3767	0.0087351
SLIT2	4	-0.29476	0.61591	-0.46067	0.67748	1.1011	0.13542	1.7712	0.038263
SLN	11	-0.39052	0.65192	0.23903	0.40554	0.92535	0.17739	0.82804	0.20382
SNCA	4	-0.15688	0.56233	1.2025	0.11458	-1.3377	0.9095	-0.93534	0.82519
SORCS1	10	0.22351	0.41157	-0.11947	0.54755	0.84101	0.20017	0.70685	0.23983
SOSTDC1	7	-0.076164	0.53036	0.53796	0.2953	0.072561	0.47108	0.18556	0.42639
SPTB	14	-2.0647	0.98052	0.35874	0.3599	-1.7576	0.96059	-0.79608	0.78701
SYT2	1	-0.44153	0.67059	0.76635	0.22173	0.9477	0.17164	0.80387	0.21074
TENM3	4	0.36845	0.35627	1.8833	0.029829	-0.30289	0.61901	-1.3159	0.9059
TMEFF2	2	0.96917	0.16623	0.35407	0.36164	-2.0342	0.97903	0.80392	0.21072
TPBG	6	-2.7065	0.9966	0.93122	0.17587	-0.28249	0.61122	-1.5772	0.94263
TWIST1	7	0.42991	0.33363	0.81407	0.2078	0.074774	0.4702	0.84682	0.19855

Supplementary Table 5. Association results for 56 genes in relation to word reading ability in 33,959 individuals, and dyslexia in 51,800 adults who reported having a diagnosis versus 1,087,070 controls

Trait	56-gene set association P value (GAUSS)
Word reading ability	0.55
Dyslexia	6.22E-09

		Word reading		Dyslexia	
GENE	Chromosome	Z	P	Z	P
AKR1C1	10	-0.68427	0.7531	-0.35201	0.63759
ATP2B2	3	-0.12062	0.548	1.6438	0.050108
BHLHE22	8	1.1984	0.11538	2.2312	0.012834
BMPER	7	-0.66136	0.74581	-1.5762	0.94251
BRINP1	9	0.88778	0.18733	1.8886	0.029472
BRINP3	1	-0.4192	0.66246	2.1302	0.016579
C3orf80	3	0.035825	0.48571	0.23086	0.40871
CALB1	8	-0.13798	0.55487	1.3123	0.094705
CASQ1	1	-1.0073	0.8431	0.04095	0.48367
CDH10	5	0.10859	0.45676	3.8645	5.57E-05
CEP83	12	-0.64322	0.73996	0.071542	0.47148
COL5A2	2	-0.3595	0.64039	1.3978	0.081086
CRYBB1	22	0.19627	0.4222	-0.15063	0.55987
DAB1	1	1.2541	0.1049	4.1955	1.36E-05
DIAPH1	5	0.48508	0.31381	2.2914	0.010972
EML1	14	-0.75145	0.77381	0.20133	0.42022
FBXO32	8	0.19406	0.42306	2.2524	0.012148
GABRD	1	-0.25609	0.60106	3.9892	3.31E-05
GAP43	3	1.5448	0.061197	0.67529	0.24975
GATB	4	2.2373	0.012635	6.6162	1.84E-11
GPR26	10	0.72976	0.23277	1.8978	0.02886
HBQ1	16	-1.8774	0.96977	1.1	0.13567
KCNH5	14	-0.31839	0.62491	2.014	0.022004
KIRREL3	11	0.24821	0.40198	2.7943	0.002601
LAMP5	20	0.023832	0.49049	1.3555	0.087631
LMO3	12	1.5216	0.064055	1.0428	0.14852
LMO4	1	-0.59703	0.72476	1.5754	0.057587
MET	7	-0.75768	0.77568	0.97615	0.16449
METTL21C	13	0.26921	0.39389	0.3732	0.3545
MYO16	13	-1.251	0.89454	0.84705	0.19848
NDST3	4	0.022425	0.49105	0.63741	0.26193
NEFH	22	-0.31447	0.62342	2.1206	0.016978
NELL1	11	-0.95908	0.83124	0.064856	0.47414
NELL2	12	1.5609	0.059278	0.086892	0.46538
OXR1	8	0.28837	0.38653	3.1456	0.000829
PDE4A	19	0.99804	0.15913	-0.94477	0.82761
PENK	8	-0.06196	0.5247	0.62349	0.26648
PRDM8	4	-0.20489	0.58117	-1.9316	0.9733
PRSS12	4	-0.09845	0.53921	-0.06274	0.52501
PTPRK	6	1.4828	0.069069	0.45383	0.32497
RSPO2	8	-0.45078	0.67393	1.0437	0.14832
RXFP1	4	-0.31035	0.62185	-1.0796	0.85984
RYR3	15	0.62454	0.26614	0.86895	0.19244
SDR16C5	8	1.8186	0.034486	-1.0081	0.8433
SLIT1	10	0.66125	0.25423	3.637	0.000138
SLIT2	4	1.5444	0.06125	1.8782	0.030175
SLN	11	-0.53487	0.70363	1.2544	0.10485
SNCA	4	0.19348	0.42329	1.9789	0.023914
SORCS1	10	0.4542	0.32484	0.84541	0.19894
SOSTDC1	7	0.82198	0.20555	0.74645	0.2277
SPTB	14	-0.47615	0.68302	0.63153	0.26385
SYT2	1	-0.19551	0.5775	1.388	0.082574
TENM3	4	1.0063	0.15713	2.5035	0.006149
TMEFF2	2	0.064835	0.47415	1.4136	0.078734
TPBG	6	0.6095	0.2711	-0.33463	0.63105
TWIST1	7	1.4555	0.072767	1.3322	0.09139

Supplementary Table 6. Association results for 56 genes based on GWAS studies of autism (46,350 individuals) and schizophrenia (82,315 individuals)

Trait	56-gene set association P value (GAUSS)
Autism	0.03
Schizophrenia	6.31E-09

		Autism		Schizophrenia	
GENE	Chromosome	Z	P	Z	P
AKR1C1	10	0.91593	0.17985	-0.30087	0.61824
ATP2B2	3	1.3295	0.091843	3.7806	7.82E-05
BHLHE22	8	0.089558	0.46432	2.4974	0.006255
BMPER	7	1.6607	0.048384	1.3343	0.091057
BRINP1	9	-1.0764	0.85912	3.3843	0.000357
BRINP3	1	-0.09076	0.53616	2.1784	0.014688
C3orf80	3	0.22443	0.41121	0.85223	0.19704
CALB1	8	-0.37427	0.6459	2.3697	0.0089
CASQ1	1	1.3886	0.082472	2.6754	0.003732
CDH10	5	1.2096	0.11322	2.6106	0.004519
CEP83	12	1.6027	0.054506	0.44977	0.32644
COL5A2	2	0.34957	0.36333	0.83737	0.20119
CRYBB1	22	-0.74768	0.77267	2.8868	0.001946
DAB1	1	-0.38297	0.64913	1.3181	0.093735
DIAPH1	5	-2.733	0.99686	0.35956	0.35959
EML1	14	-0.33185	0.63	2.4178	0.007808
FBXO32	8	0.48879	0.31249	0.41645	0.33854
GABRD	1	1.4751	0.070093	0.48661	0.31327
GAP43	3	-0.71466	0.76259	0.52799	0.29875
GATB	4	2.7997	0.002558	0.71414	0.23757
GPR26	10	0.21734	0.41397	0.2434	0.40385
HBQ1	16	-0.14839	0.55898	0.20478	0.41887
KCNH5	14	2.4817	0.006538	1.7676	0.038565
KIRREL3	11	0.36091	0.35908	3.4017	0.000335
LAMP5	20	0.79719	0.21267	-0.79664	0.78717
LMO3	12	0.73076	0.23246	0.76742	0.22142
LMO4	1	-0.79203	0.78583	0.16823	0.4332
MET	7	0.43715	0.331	0.82669	0.20421
METTL21C	13	-0.49981	0.69139	2.2979	0.010785
MYO16	13	-0.17224	0.56837	2.1987	0.013948
NDST3	4	1.2393	0.10762	3.0476	0.001154
NEFH	22	1.1643	0.12215	0.1326	0.44725
NELL1	11	0.17971	0.42869	0.83458	0.20198
NELL2	12	-0.42087	0.66307	0.90846	0.18182
OXR1	8	0.66017	0.25457	-0.07239	0.52885
PDE4A	19	-0.08674	0.53456	-0.21824	0.58638
PENK	8	0.85674	0.19579	-0.11788	0.54692
PRDM8	4	-1.3509	0.91163	2.7843	0.002683
PRSS12	4	1.4996	0.06686	2.0976	0.01797
PTPRK	6	1.2304	0.10927	0.48908	0.31239
RSPO2	8	-0.21467	0.58499	1.1252	0.13025
RXFP1	4	1.4665	0.07126	-0.11067	0.54406
RYR3	15	-0.38363	0.64937	1.6586	0.048597
SDR16C5	8	0.81302	0.2081	-0.08437	0.53362
SLIT1	10	-0.34338	0.63434	1.0055	0.15732
SLIT2	4	1.3685	0.085579	1.3108	0.094959
SLN	11	0.27532	0.39153	1.1541	0.12423
SNCA	4	1.7034	0.044247	2.2444	0.012404
SORCS1	10	0.99694	0.1594	1.7554	0.039596
SOSTDC1	7	-0.07157	0.52853	1.3362	0.090741
SPTB	14	1.1743	0.12013	0.3527	0.36216
SYT2	1	1.9009	0.028656	0.24676	0.40255
TENM3	4	0.94635	0.17198	3.6258	0.000144
TMEFF2	2	-0.58483	0.72067	1.0521	0.14637
TPBG	6	0.40817	0.34157	1.9653	0.024692
TWIST1	7	0.20742	0.41784	1.0213	0.15356
